# Unraveling Genetic Modifiers in the *Gria4* Mouse Model of Absence Epilepsy

**DOI:** 10.1371/journal.pgen.1004454

**Published:** 2014-07-10

**Authors:** Wayne N. Frankel, Connie L. Mahaffey, Tracy C. McGarr, Barbara J. Beyer, Verity A. Letts

**Affiliations:** The Jackson Laboratory, Bar Harbor, Maine, United States of America; Stanford University School of Medicine, United States of America

## Abstract

Absence epilepsy (AE) is a common type of genetic generalized epilepsy (GGE), particularly in children. AE and GGE are complex genetic diseases with few causal variants identified to date. *Gria4* deficient mice provide a model of AE, one for which the common laboratory inbred strain C3H/HeJ (HeJ) harbors a natural IAP retrotransposon insertion in *Gria4* that reduces its expression 8-fold. Between C3H and non-seizing strains such as C57BL/6, genetic modifiers alter disease severity. Even C3H substrains have surprising variation in the duration and incidence of spike-wave discharges (SWD), the characteristic electroencephalographic feature of absence seizures. Here we discovered extensive IAP retrotransposition in the C3H substrain, and identified a HeJ-private IAP in the *Pcnxl2* gene, which encodes a putative multi-transmembrane protein of unknown function, resulting in decreased expression. By creating new *Pcnxl2* frameshift alleles using TALEN mutagenesis, we show that *Pcnxl2* deficiency is responsible for mitigating the seizure phenotype – making *Pcnxl2* the first known modifier gene for absence seizures in any species. This finding gave us a handle on genetic complexity between strains, directing us to use another C3H substrain to map additional modifiers including validation of a Chr 15 locus that profoundly affects the severity of SWD episodes. Together these new findings expand our knowledge of how natural variation modulates seizures, and highlights the feasibility of characterizing and validating modifiers in mouse strains and substrains in the post-genome sequence era.

## Author Summary

Absence seizures - also known as “petit-mal” - define a common form of epilepsy most prevalent in children, but also seen at other ages, and in related diseases such as juvenile myoclonic epilepsy. Absence seizures cause brief periods of unconsciousness, and are accompanied by characteristic abnormal brain waves called “spike-wave discharges” (SWD) due to their appearance in the electroencephalogram (EEG). Although few genes are known for human absence seizures, perhaps because the underlying genetics are complex, several laboratory rodent models exist, including one caused by mutation of a gene called *Gria4*. While studying *Gria4*, we noticed that a mouse strain called C3H can suppress or enhance the frequency and severity of *Gria4*-associated SWD in a perplexing manner; such effects are generally attributed to “modifier” genes. Here we identify a novel modifier – called “pecanex-like 2”, or *Pcnxl2* for short – that reduces the severity of SWD in the C3H substrain in which the *Gria4* mutation originally arose. This finding directed us to use of related substrains to locate additional modifiers, one of which has an even more profound effect on SWD episodes. Modifier genes, nature's way of controlling seizure severity, are promising targets for better understanding seizure mechanisms and potential new therapies in the future.

## Introduction

Laboratory mouse strains are well known to vary in their susceptibility to convulsive seizures, including acute experimentally induced seizures [1], [2], [3], [4], [5], [6], and spontaneous seizures induced by genetic mutation [7], [8], [9], [10], [11], [12], [13]. Most of the known strain effects have been for convulsive seizures, where motor manifestations are obvious, including one modifier identified to date (*Kcnv2* as a modifier of *Scn2a1* ^Q54^ induced convulsive seizures [13]. There has been less attention to such effects in non-convulsive phenotypes, such as absence epilepsy, where the seizures lack a convulsive element. Nevertheless, strain differences were noted for three different genes that cause absence seizures when mutated – *Scn8a* [14]), *Gabrg2* [15] and *Gria4* [16], and for at least two the C3H strain generally worsens the absence seizure phenotype, compared with the relatively protective strain C57BL/6J (B6J).

For absence seizures caused by *Gria4* mutation, the C3H strain has been a paradox. Each of three very closely-related C3H/He substrains has a similar level of spontaneous spike-wave discharges (SWD) - the distinctive electroencephalographic hallmark of absence seizures in human and in animal models - but only one substrain, C3H/HeJ (HeJ), carries a *Gria4* mutation. This mutation is caused by an intracisternal A-particle retrotransposon (IAP) insertion in *Gria4* [16], [17], [18]. Although genetic and later functional analysis proved that *Gria4* is the cause of these seizures in HeJ [19], two other C3H/He substrains that lack this mutation still have appreciable SWD. At least one of these strains was shown to have a polygenic etiology, with no indication of any effect from proximal Chr 9 where *Gria4* resides [18]. The further surprise was when HeJ mice were outcrossed to other strains - even to another C3H substrain, C3HeB/FeJ (FeJ), that does not have frequent SWD - about half of the next generation *Gria4* deficient progeny had significantly higher SWD incidence than HeJ itself [16]. Together these results suggested the model whereby the HeJ substrain has both the initial seizure-causing *Gria4* mutation, and also a mitigating or protective mutation, without which the seizures would be much more severe. The model also suggests that C3H strains in general have a high baseline susceptibility to absence seizures, compared with strains such as C57BL/6.

IAP insertions like the element in *Gria4* are known to cause deleterious mutant phenotypes by reducing RNA expression of the target gene, and the vast majority of spontaneous IAP insertion mutations in mice have arisen in the C3H strain family (reviewed in [20]). While searching for genetic markers to distinguish C3H substrains for analysis of the putative HeJ suppressor, we discovered extensive IAP retrotransposition among C3H substrains. One of the HeJ substrain-private IAP insertions resides in the same chromosomal region as an epistatic modifier of *Gria4* absence seizures mapped to Chr 8 [17]. Here we report that this Chr 8 IAP is inserted in the previously unstudied *Pcnxl2* (pecanex-like 2) gene, affecting its RNA expression. We further use TALEN mutagenesis to create new *Pcnxl2* alleles, confirming that *Pcnxl2* loss of expression is responsible for mitigating *Gria4* seizures of HeJ mice, accounting for the substrain difference. We also mapped modifiers that differ between C3H and B6J, and show that one – *G4swdm1* – has a profound effect on seizure severity. The identification of the first absence seizure modifier *Pcnxl2* provides significant traction to the complex genetics of absence seizures in the C3H strain family and possible new mechanisms for mitigating disease.

## Results

### Congenic strains highlight strain background effects on seizures

Prior studies of *Gria4*-deficient mice showed significant genetic background influence on the incidence and duration of spike-wave discharges (SWD) between C57BL/6J (B6J) and C3H strains [16], [17], whether the natural *Gria4* ^spkw1^ allele (abbreviated hereafter as Gria4^IAP^) or the engineered knockout *Gria4* ^tm1Dgen^ (*Gria4* ^KO^). While mapping the main phenotype to *Gria4* in a backcross between C3H/HeJ (HeJ) and B6J, about half of the progeny were noted to have significantly more SWD than HeJ itself, and an epistatic modifier putatively due to this effect was mapped to distal Chr 8 [16], [17]. Interestingly, a similar phenomenon from crosses between HeJ and C3HeB/FeJ (FeJ) suggested this was due to a substrain difference [16], [17]. To confirm this, we backcrossed *Gria4* ^IAP^ allele from HeJ to FeJ and examined SWD in this congenic pair and compared to B6J-*Gria4* ^KO^ for reference. FeJ-*Gria4* ^IAP^ had significantly more frequent and longer SWD than HeJ or B6J-*Gria4* ^KO^ (Table 1). These results indicate that HeJ has a suppressor allele(s) not present in FeJ. It also suggest that further modifiers differ between FeJ and B6J strains.

**Table 1:** Genotypes and features of inbred and congenic strains used in this study.

	Allele			SWD incidence (avg. per hr ± sem)	SWD length (avg. sec ± sem)	N
Strain (abbr.)	*Gria4*	*Pcnxl2*	*G4swdm1*			
C3H/HeJ (HeJ)	IAP	IAP	susceptible	11.1±1.5	2.8±0.19	9
C3Heb/FeJ (FeJ)	WT	WT	susceptible	<2	<1	[ref 17]
C57BL/6J (B6J)	WT	WT	resistant	0	0	[ref 17]
FeJ.HeJ-*Gria4* ^spwk1^ (FeJ-*Gria4* ^IAP^)	IAP	WT	susceptible	52.1±3.4	4.2±0.2	14
B6.129-*Gria4* ^tm1Dgen^ (B6J-*Gria4* ^ko^)	KO	WT	resistant	13.3±4.0	2.3±0.28	8

### Extensive IAP insertion variation in C3H substrains

The HeJ suppressor is a challenge to pursue by classical recombination mapping because of the paucity of genetic markers between FeJ and HeJ substrains, which split into separate lines around 1950 [21]. However, C3H mice are known to have frequent spontaneous germline IAP retrotransposition, with *de novo* insertions accounting for a significant fraction of the spontaneous mutation load in C3H (see review by [20]). Moreover, almost all *de novo* IAP insertions, including *Gria4* ^IAP^, are of a particular IAP subtype - IAP-1Δ1 - containing a characteristic in-frame 1.2 kb deletion of the gag-pol fusio gene [22]. Sequence similarity search of the B6J mouse genome assembly with an oligonucleotide sequence spanning the IAP-1Δ1 deletion detected approximately 200 independent genomic sites, whereas the same region from full-length IAP detected over 700 sites (data not shown), suggesting the feasibility of direct hybridization approaches to identify substrain-specific *de novo* IAP-1Δ1 insertions.

**Figure 1:**
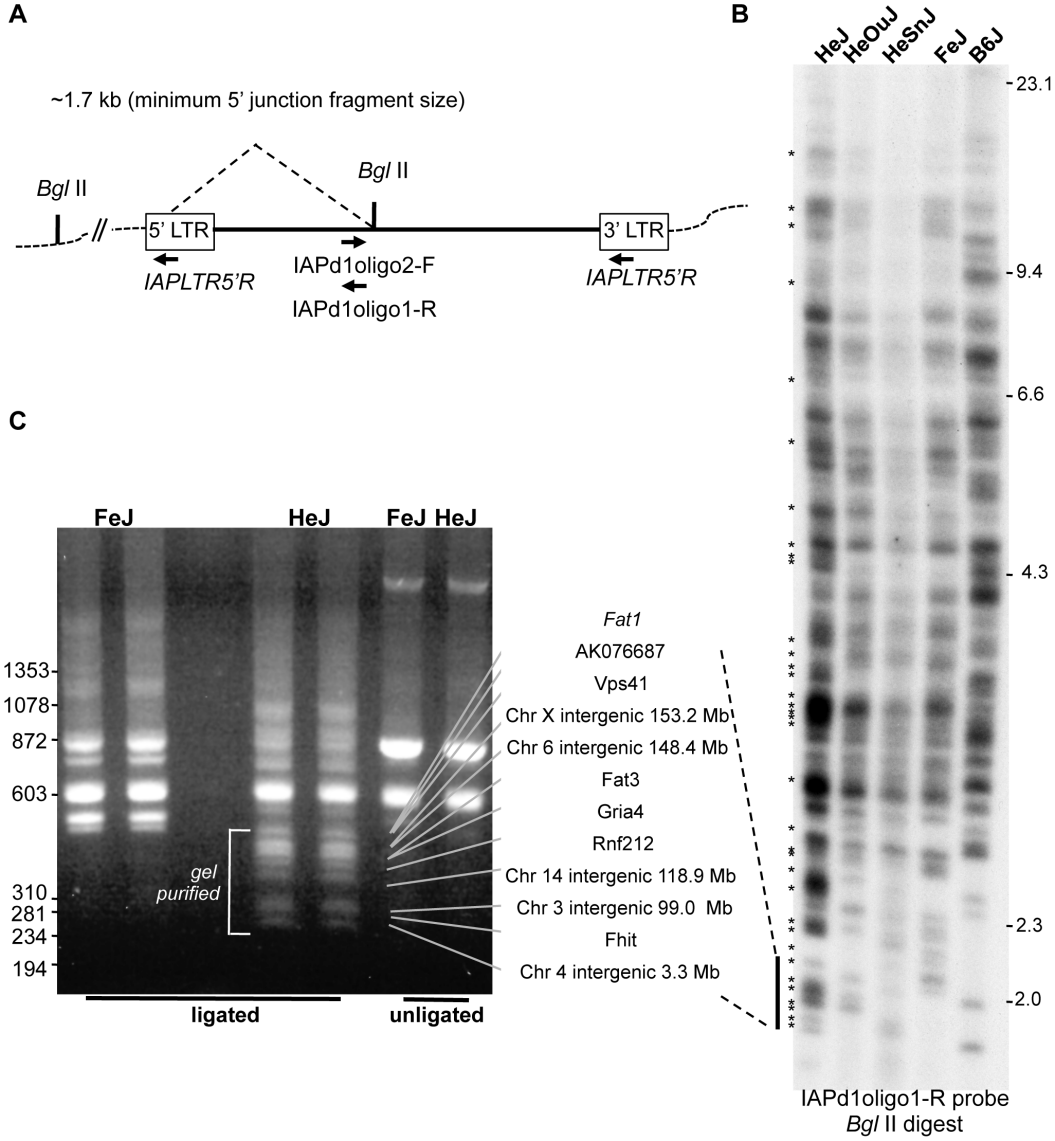
IAP-1Δ1 element diversity in C3H substrains. A. Strategy for detection of IAP-1Δ1 elements. For detection of host-IAP-1Δ1 5′ *Bgl*II-restricted junction fragments by dried gel hybridization, a 31-nt oligonucleotide hybridization probe (IAPd1oligo1-R) was designed to straddle the previously described 1.2 kb IAP-1Δ1 element common deletion [22]. For cloning of IAP-1Δ1 elements by inverse PCR, a complementary oligonucleotide (IAPd1oligo2F) was designed to pair with an oligonucleotide specific for the IAP LTR (IAPLTR5′) for amplification of circularized genomic *Bgl*II fragments. B. Dried gel detection of IAP-1Δ1 5′ junction fragments from C3H substrains (HeJ, HeOuJ, HeSnJ, FeJ) plus the outlier B6J. Bands estimated as unique to HeJ are indicated with asterisks on the left. C. Example of an inverse PCR experiment from HeJ and FeJ substrains showing *Bgl*II restricted genomic DNA (two lanes each), unligated controls (one lane each), and location of apparent HeJ-specific bands prepared. for cloning and Sanger sequencing. In additional experiments not shown, higher molecular weight *Bgl*II restricted fragments were similarly processed. Also shown with connecting lines are locus identities for specific bands after sequencing, the sizes of which correspond to the junction fragment lengths determined from the sequence, and dashed lines indicate the corresponding region from the dried gel, for the purpose of highlighting the similar banding pattern (*N.b.* the absolute sizes of fragments in panel C are smaller because the portion of the IAP genome between primers is expectedly absent from the inverse PCR product). Sizes of *Hind*III-digested bacteriophage lambda DNA are shown on the right, of panel B, and *Hae*III-digested bacteriophage phi-X DNA markers are shown at the left of panel C.

To determine whether C3H substrains vary significantly in IAP-1Δ1 content, we designed an oligonucleotide specific for the IAP-1Δ1 1.2 kb common deletion to examine proviral-host DNA junction fragments by direct dried gel hybridization and to subclone them by inverse PCR (Fig. 1A). Although it is unlikely that any visible ‘band’ in the direct hybridization represents a single IAP insertion, from the differential pattern– best observed in the 2 kb range - there are ample variation between four substrains examined; with HeJ appearing to have the highest load including at least 20 HeJ-specific bands evident (Fig. 1B). Inverse-PCR was used to identify and clone select IAP-1Δ1 – host junction fragments from FeJ and HeJ. A representative experiment to identify lower molecular weight junction fragments revealed a banding pattern reminiscent of the gel hybridization, and recapitulates the corresponding paucity of bands in this region in the FeJ strain (Fig. 1C). From this and other experiments, bands were excised, cloned and junction fragments sequenced, and PCR assays were developed ultimately leading to identification of at least 26 insertion-host junction fragments that are present in the HeJ substrain and absent from FeJ (Table 2). 9 are intergenic, but the majority are in introns of known or unclassified genes (Table 2). Most HeJ insertions we cloned were ultimately identified in the recent retrotransposon-mining of the whole genome sequence of HeJ and other mouse strains from the Sanger Mouse Genomes Project [23], although substrain specificity was not ascertained in that study.

**Table 2:** C3H/HeJ IAP-1Δ1 insertions absent from C3HeB/FeJ.

Chr	Position (m38)	Assay	Gene	Gene Forward Primer (5′-3′)	Gene Reverse Primer (5′-3′)
1	6394695	c1-6.4	*Fam150a*	GTGCCTGGATCTGGAGTTGT	AAATTCACAGTTCAAATTAATGCAA
2	10357719	c2_10.2	*AK076687*	AGATCTGATTACTGAGGGC	CACATGTCTGTCTGTCTGTTTGAG
3	14164948	c3-14.2	*Ralyl*	TTCACAAAAATATTCTAGTAAGGCTCA	TCACATCCTTCTCACAGACTCAG
4	45559646	c4-45	intergenic	TGGCTTGAAACTCAGCAAGA	ATATGGCCCACGCTAGTCTC
4	104102700	c4-103.8	*Dab1*	GGGTACTTACTTATTCCTGAACATGC	AAAGAGCATTATAGGTGCATTCC
5	20214596	c5-19	*Magi2*	CTGCCATCTTCAAGTACTCAGG	TGCTCACATTTGATGCTTTGA
5	31659391	c5-31.9a	*Rbks*	TTCAGAGGAAAATGGGTAGGAA	GCAAAGATTCCTTCTTTCTCTGTT
6	38291929	c6-38.2	*Zc3hav1l*	TCCCTCCTCTCCTTTGATCC	TCTGTACCCACTCATCCTGTTG
6	148468196	c6-148	intergenic	GCATAGCCCAGCATGACTCT	TGGATAGCAGGGTGAACTT
8	11527565	c8-11.5	*Cars2*	TTGCTGTCCCAGTCAGAAGA	CAGGCAAATGACCAGGAAGT
8	44964823	c8-46	*Fat1*	AGAGGCAGAAAGAGCGTGAG	GGGTTTTAATTGTCTGTGTTATTGC
8	84219375	c8-86	*Zswim4*	AAAGGTGAGGGCAGTGACAG	CAGGTGTGGCAGAACATGAG
8	125820583	c8-128.3	*Pcnxl2*	AGCGATGAGGACTGTGGTTT	CGAGCCCTTCAGCTACTCAC
10	83109544	c10-82.6	*Chxt11*	GGGAGTCAGGTAGCAGAGTGA	GCCAGGCAAACTGTGAGACT
11	65662429	c11-65.5	intergenic	TTCCAGGCTGTGCGTATAAA	TCAACCAGCTGTTAGGTCAGC
11	81057142	c11-80.9	*Asic2*	CCAAAGAGCTTGGGTTCAAA	TGGAAACCAGGAAGGGTATG
13	16513994	c13-16.6	intergenic	TTTGCTTTGTTATGGATTGTTCA	GGGCCAAAGACTAACTTTTAACC
13	53603004	c13-53.7	intergenic	CGCAATTCCTAAACCTGGAA	TGTGGACTGCTTGGTAGCAT
13	83101738	c13-83.2	*2310067P03Rik*	GCGTGACAAGATGGCTCATA	CATCAGATGAATACTTTTTATCATTCC
14	9640485	c14-10.4	*Fhit*	GGCATGAAAACAAACATCCA	GCATCATTCAATCAGCTATAGGG
14	118932598	c14-119	intergenic	ACAATACAGCTCCCCCACAG	AAGCTGTCCTCCGACTTTCA
16	83756907	c16-83.8	intergenic	CAGAAATTGGCCAGGAAAGA	AAAGAATAATGATCTTTCAGTTCAGG
17	22747296	c17-22	intergenic	CAGAGAGCACATGCCAAAAA	TTTGGGTGAGAATACTCTTCCTG
17	90348848	c17-90.7	*Nrxn1*	CAGGAGCTGTTATGAAAAGCAA	CATGGCACAGATGGGAGAAT
X	6519183	cX-6.2	*Shroom4*	GGCTGCTTTAGCTGCCATA	TGAAGGCTCTTGAGGAAGGA
X	153202745	cX-149	intergenic	CTGGAAGGCTCCTCACAAAG	GGCCCATGTGTATGTGGTTT
IAP^a^	na	IAP5′LTR-F	IAP	GGCTCATGCGCAGATTATTT	

a To detect both IAP insertion allele and the pre-insertion allele, the IAP5′LTR-F oligonucleotide is used in a 3′-primer assay with both the locus-specific forward and reverse primers. In cases where the product sizes of both alleles were identical, the IAP5′LTR-F+gene-F (or gene -R, depending upon IAP orientation) for the insertion allele and gene -F+gene -R primers are run separately for the pre-insertion allele.

### Reduced expression of *Pcnxl2* due to a substrain-specific IAP insertion

One HeJ-specific IAP insertion resides at 128.3 Mb on Chr 8, in the same general region of the previously mapped epistatic modifier of *Gria4* SWD [17]. This insertion was not seen in any of the 17 inbred mouse strains for which genome sequence is available [23], and the PCR assays used to identify it (Table 2) determined that it was also absent from C3HeB/FeJ (FeJ), C3H/HeOuJ and C3H/HeSnJ substrains (data not shown). This IAP is integrated in the 5′-LTR-3-′LTR orientation in intron 19 of *Pcnxl2*, the gene that encodes pecanex-like 2. *Pcnxl2* is one of three mammalian paralogs of *Drosophila melanogaster* pecanex, a neurogenic gene about which little is known, but was recently suggested to be part of the notch signaling pathway in the endoplasmic reticulum [24], [25]. In adult mouse brain, *Pcnxl2* is expressed highest in the hippocampal pyramidal cell layer, it is also expressed prominently in the area of cerebral cortex corresponding to layer V, and sparsely in the reticular thalamic nucleus (Allen Brain Atlas: see http://mouse.brain-map.org/experiment/show/70239051). As the latter two regions are key in abnormal cortico-thalamic oscillations associated with absence seizures generally and specifically in *Gria4* mutants [19], *Pcnxl2* is a suitable candidate for the suppressor.

To determine whether the IAP insertion affects *Pcnxl2* gene expression, we initially examined *Pcnxl2* transcript expression in brain from public datasets, comparing HeJ to other inbred strains. From inspecting publically available mouse strain hippocampal microarray (http://www.genenetwork.org) and in the Sanger Center's whole-brain RNAseq (ftp://ftp-mouse.sanger.ac.uk/current_rna_bams), we noted an HeJ-specific drop in relative expression or abundance of exons downstream of exon 19 (data not shown). To examine this between HeJ and FeJ substrains, by using quantitative RT-PCR we confirmed that *Pcnxl2* expression across intron 19 is indeed lower in HeJ compared to FeJ (Table 3, compare sample IDs 1–2).

**Table 3:** Quantitative RT-PCR in *Pcnxl2* mutants.

Target transcript	Strain	*Pcnxl2* genotype	Fold-diff a	ΔCt avg	±SD	Sample ID
*Pcnxl2*	HeJ (*Gria4* ^IAP^)	IAP/IAP		8.37	0.1	1
*“ ”*	FeJ-*Gria4* ^IAP^	+/+	2.0	7.36	0.12	2
*“ ”*	FeJ-*Gria4* ^IAP^	FS2		8.09	1.49	3
*“ ”*	FeJ-*Gria4* ^IAP^	+/+	3.5	6.29	0.58	4
*“ ”*	FeJ-*Gria4* ^IAP^	FS1		8.85	0.38	5
*“ ”*	FeJ-*Gria4* ^IAP^	+/+	1.9	7.92	0.05	6
*“ ”*	FeJ-*Gria4* ^IAP^	FS3		8.44	0.23	7
*“ ”*	FeJ-*Gria4* ^IAP^	+/+	2.5	7.12	0.3	8
*Gria4*	FeJ-*Gria4* ^IAP^	FS1		9.17	0.66	9
*“ ”*	FeJ-*Gria4* ^IAP^	+/+	1.2	8.93	0.65	10
*“ ”*	FeJ-*Gria4* ^IAP^	+/+		7.03	0.1	11
*“ ”*	FeJ (*Gria4* ^+^)	+/+	8.8	3.9	0.17	12
*Pcnx*	FeJ-*Gria4* ^IAP^	FS2		4.84	0.14	13
*“ ”*	FeJ-*Gria4* ^IAP^	+/+	1.1	4.69	0.17	14
*“ ”*	FeJ-*Gria4* ^IAP^	FS1		4.7	0.47	15
*“ ”*	FeJ-*Gria4* ^IAP^	+/+	1.0	4.64	0.28	16
*“ ”*	FeJ-*Gria4* ^IAP^	FS13		4.6	0.18	17
*“ ”*	FeJ-*Gria4* ^IAP^	+/+	−0.9	4.81	0.2	18

a Fold-difference for each pair of rows was calculated as 2^(top row ΔCt – bottom row ΔCt)^, and is signed relative to the bottom row (e.g. *Pcnxl2* transcript is 2.0 fold higher in FeJ-*Gria4* ^IAP^ compared to HeJ.

### TALEN alleles confirm reduced *Pcnxl2* expression modifies *Gria4* absence seizures

**Figure 2:**
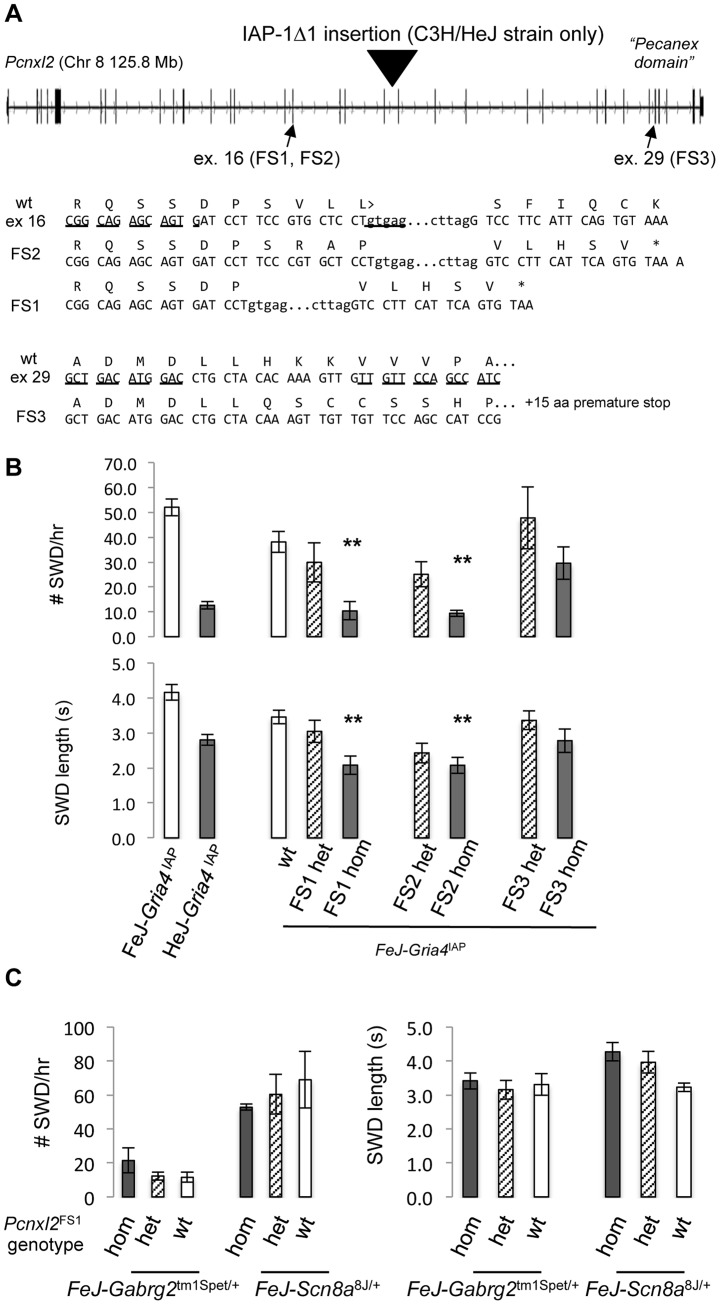
TALEN-induced frameshift alleles of *Pcnxl2* and suppression of *Gria4* SWD. A. Exon-intron structure of mouse *Pcnxl2* (from UCSC genome browser, version m38 mouse genome assembly), highlighting the position of the HeJ-specific IAP-1Δ1 insertion in intron 19, the two exons (exon 16 and exon 29, respectively) targeted for TALEN mutagenesis and the wildtype FeJ sequence of each together with the sequence of the respective frameshift alleles in TALEN mutations A+1 (FS2), A−11 (FS1), and B−2 (FS3). The asterisk indicates a premature translational stop codon in each mutant allele. B. Spike-wave discharge (SWD) incidence (top) and length (bottom) for parent FeJ-*Gria4* ^IAP^ congenic and HeJ-*Gria4* ^IAP^ inbred strain colonies, compared with littermate wildtype (wt), heterozygous or homozygous respective TALEN mutations created and maintained on the isogenic FeJ-*Gria4* ^IAP^ congenic strain background. Asterisks indicate where homozygous mutant genotypes were significantly different (*p*<0.01) from wildtype control. Sample sizes: FeJ-*Gria4* ^IAP^ (9), HeJ- *Gria4* ^IAP^ (18), wt (16), FS1 het (6), FS1 hom (6), FS2 het (9), FS2 hom (9), FS3 het (6), FS3 hom (6). Error bars are SEM. C. Spike-wave discharge (SWD) incidence (left) and length (right) for littermate TalA-11 genotypes (hom, het, wt) isogenic on the FeJ strain, in double mutant combination with *Gabrg2* ^tm1Spet^ or *Scn8a* ^8J/+^ from a congenic FeJ strain background. None of the *Pcnxl2* mutant allele homozygotes were significantly different from the other *Pcnxl2* genotypes for either mutation or SWD measure. Sample sizes: *Gabrg2* ^tm1Spet^ (3 hom, 12 het, 2 wt); *Scn8a* ^8J/+^ (2 hom, 4 het, 2 wt).

To confirm that *Pcnxl2* encodes the *Gria4* SWD suppressor, we used TALEN mutagenesis [26] in the FeJ-*Gria4* ^IAP^ congenic strain to create *Pcnxl2* frameshift mutations (Fig. 2A). Two *Pcnxl2* exons were targeted separately: exon 16, roughly in the middle of the gene, and exon 29 nearer the 3′ end, the latter containing the so-called “pecanex” domain – a conserved domain of unknown function shared among pecanex paralogues. Three in 106 liveborn mice contained germline mutations and each created a frameshift allele that resulted in a premature translational stop codon (Figure 2).

To test the effect on SWD, EEG was examined in FeJ-*Gria4^IAP^* homozygotes carrying each of the three *Pcnxl2* mutations (A−11, A+1 and B−2, hereafter referred to as FS1 – frameshift 1, FS2 and FS3, respectively). *Pcnxl2* ^FS1^ and *Pcnxl2* ^FS2^ mutations significantly lowered SWD incidence (Fig. 2B) and length (Fig. 2C), showing an additive effect across genotypes. The homozygotes were also slightly more suppressed than HeJ with its natural *Pcnxl2* ^IAP^ allele. An examination of *Pcnxl2* transcripts by qPCR showed decreased (between 2 and 3.5-fold; Table 3, compare sample ID's 3–4, 5–6, 7–8) but not eliminated expression in each homozygous genotype; this is expected as frameshift mutations in middle exons often cause nonsense-mediated decay. However, the *Pcnxl2* ^FS3^ allele did not decrease SWD as effectively as the others (Fig. 2B, C). Since commercial antisera to PCNXL2 are not effective in mouse brain (data not shown), we cannot know whether this is because a partial or truncated protein is still made and has some residual function, or whether nonsense-mediated decay was less complete. Regardless, the effect of at least two independent new *Pcnxl2* alleles on SWD, combined with transcript reduction shows that *Pcnxl2* encodes the suppressor of *Gria4* SWD.

### *Pcnxl2* deficiency does not have a large effect on *Scn8a* ^8J^ or *Gabrg2* ^tm1Spet^ mutant SWD

To determine whether *Pcnxl2* deficiency can affect SWD in other absence seizure mouse models, we examined double mutant genotypes between FeJ-*Pcnxl2* ^FS1^ and either *Scn8a* ^8J^ [14], or *Gabrg2* ^tm1Spet^ [15], each of which encodes a dominant, SWD-causing missense mutation in the respective ion channel gene. *Pcnxl2* deficiency had no significant effect on SWD on either of these mutants (Fig. 2C), suggesting that *Pcnxl2* may be specific for mechanisms that involve *Gria4*. However, if *Gria4*-specific, the mechanism appears not to be on IAP mutagenesis itself, because there is no increase in *Gria4* RNA expression in *Gria4* ^IAP^, *Pcnxl2* ^FS1^ double mutants (Table 3, compare sample IDs 9–10). We also note that the *Pcnxl2* paralogue, *Pcnx*, is expressed widely in adult mouse brain, including likely overlap with *Pcnxl2* (http://mouse.brain-map.org/experiment/show/73818801), suggesting the possibility of compensation. However, the *Pcnx* transcript is not altered in any of the three new *Pcnxl2* frameshift alleles (Table 3, compare sample IDs 13–14, 15–16, 17–18), suggesting that if there is any compensation by this overlapping pecanex gene, it is not at the transcript level.

### *G4swdm1*, an inter-strain modifier of *Gria4* absence seizures

**Figure 3:**
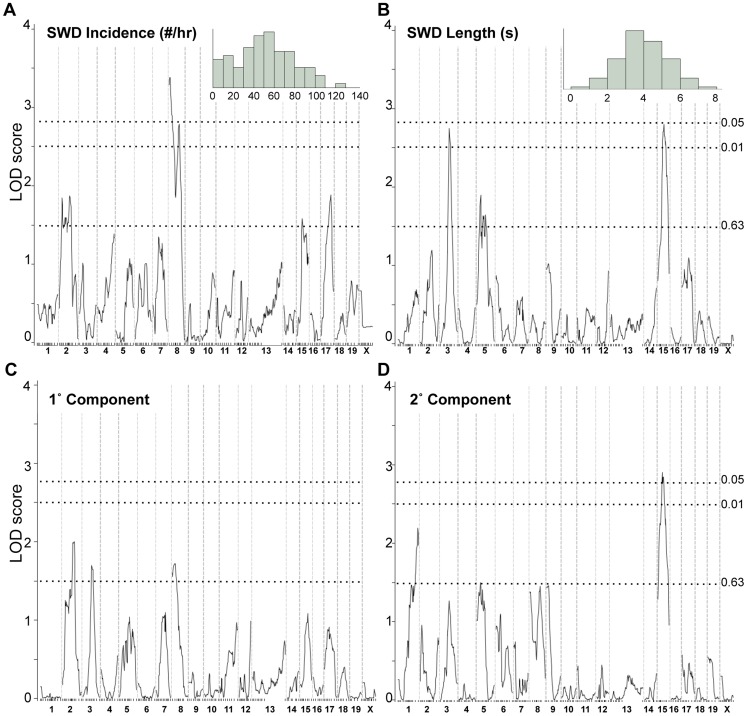
Genome scans for SWD traits in *Gria4* deficient backcross mice. Shown are genome-wide interval mapping LOD score plots from 89 backcross *Gria4* mutant mice for two observed spike-wave discharge (SWD) traits - SWD incidence (panel A), SWD length (panel B) - and their first two principal components (panels C and D). The insets of panels A and B show the frequency distribution of the respective raw traits, although for interval mapping all four traits were first rank- and quartile-normalized prior to analysis. The chromosomes are shown at the bottom, with tick marks indicating positions of individual markers within each chromosome. Dotted lines show positions of genome-wide significance thresholds determined from 1000 permutations.

The fact that *Gria4* mutant associated SWD are more pronounced when placed on the C3HeB/FeJ (FeJ) strain compared with B6J suggests additional modifiers, i.e. separate from *Pcnxl2* whose genotype does not differ between B6J and FeJ (Table 1). To pursue these, we backcrossed (FeJ-*Gria4* ^IAP^×B6J-*Gria4* ^KO^)F_1_ animals to B6J- *Gria4* ^KO^, creating 89 N_2_ mice segregating B6J vs. FeJ strain variants on a *Gria4* mutant background (either homozygous *Gria4* ^KO^ or compound heterozygous *Gria4* ^KO/IAP^) and scored their EEG for SWD incidence and length. The broad, almost continuous distribution of each raw SWD measure in this population suggests multiple factors (Fig. 3A, 3B insets). A genome-wide scan of the two SWD measures and their principal components revealed two highly significant regions (Fig. 3A, 3B): proximal Chr 8 (peak LOD, 3.4) and mid Chr 15 (peak LOD, 2.9). The Chr 15 region was significant for SWD length but negligible for incidence, and *vice versa* for Chr 8 (compare Fig. 3A with 3B), suggesting each region is primarily responsible for one of the two measures, at least in this cross. A region in mid Chr 3 was significant for SWD length only, and several suggestive peaks were observed for at least one SWD measure on Chrs 1, 2 and 5. A pairwise scan was done to look for pairwise epistatic interactions, but no significant interactions were observed (data not shown).

**Figure 4:**
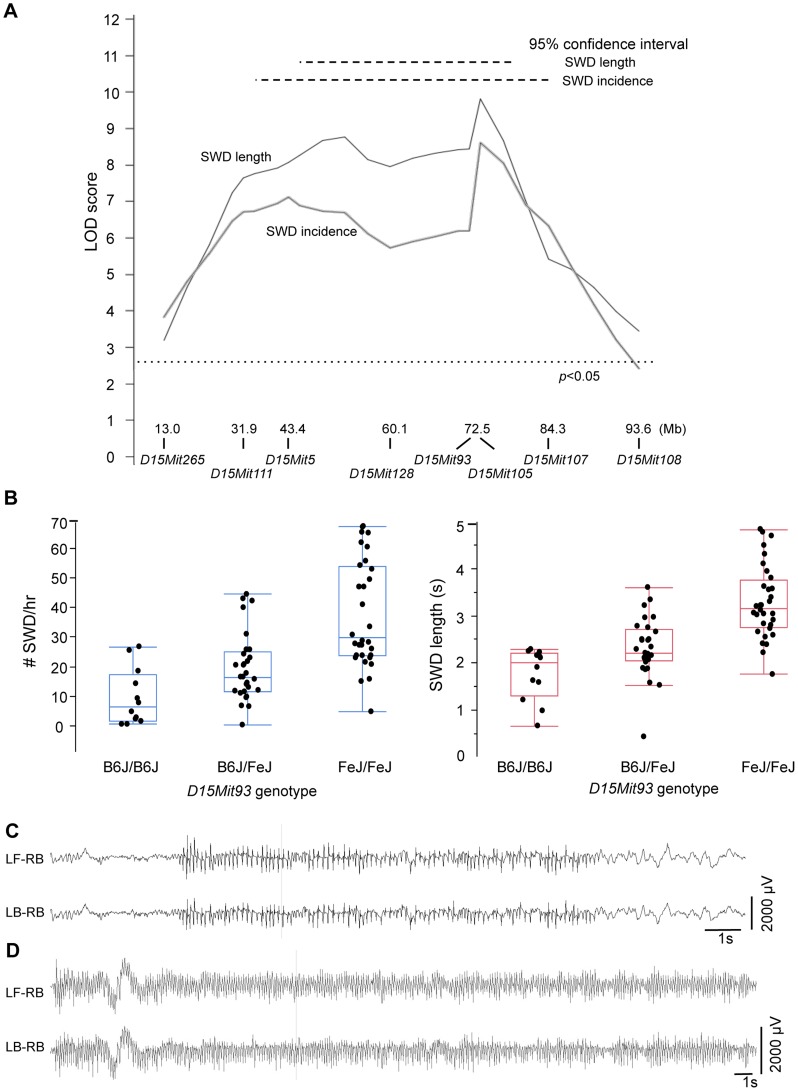
SWD phenotypes conferred by *G4swdm1*, a Chr 15 strain modifier of *Gria4* absence seizures. A. LOD score plot, similar to those in Figure 3, except only for Chr 15 in 84 N_9_F_1_ or N_10_F_1_ intercross congenic mice. At the bottom are Mb (mouse genome assembly m38) positions in of Chr 15 markers used for this analysis and the genome-wide significance threshold is also shown, as determined by 5000 permutations. B. For the marker *D15Mit93* corresponding to the peak maximum likelihood location from panel A, quartile plots with data points for average SWD incidence or SWD length from individual mice, showing additivity for both traits. C. EEG from a *Gria4* ^KO/KO^, *G4swdm1^Fe/FeJ^* double homozygote on the B6J congenic strain, showing a particularly long (15 second) SWD. D. EEG from a *Gria4* ^KO/IAP^ compound heterozygous, *G4swdm1^Fe/FeJ^* homozygous double mutant genotype on a (B6J.FeJ×FeJ)F_1_ hybrid background, showing a particularly long (46 second) SWD. For panels C and D, two of the six recorded channels are shown (LF-RB – left front, right back; LB-RB – left back, right back, corresponding to the electrode placement relative to Bregma and midline). 1 s: 1 second.

Because of the increased SWD length, we focused on validating the Chr 15 locus, now termed *G4swdm1*, for *Gria4* spike-wave discharges modifier 1. We made a congenic strain in which the middle of Chr 15 was selectively bred from FeJ into B6J-*Gria4* ^KO^, and N_9_F_1_ and N_10_F_1_ intercross mice were generated and tested. Highly significant effects were observed this time for both SWD length and incidence across the introgressed interval (Figure 4A, 4B). While *G4swdm1* was initially detected as a dominant allele, the congenic intercross reveals additivity: FeJ homozygotes experience significantly longer and more frequent SWD (Figure 4C). Although SWD length was still the leading phenotype, in the B6J background the effect of *G4swdm1* on SWD incidence was stronger than in the N_2_ population, likely reflecting additional genetic complexity. Indeed, several congenic individuals had SWD 15 s long (Fig. 4D); further, when the homozygous interval was placed together with the *Gria4* mutant genotype on a (B6J×FeJ)F_1_ hybrid background, several very striking SWD, exceeding 40 s were observed (Figure 4D). The estimated 95% confidence interval (CI) for *G4swdm1* is large, with the narrowed SWD length interval covering 27.2 Mb including 191 protein coding and 75 small RNA or unclassified genes (File S1), although the “bumpy” likelihood curve suggests the possibility of multiple modifiers. Among these genes are 27 that have 56 non-synonymous coding or potential splice altering SNPs or indels, between published C3H/HeJ and B6J genomes; further, comparison of FeJ exome sequence to HeJ revealed no similar coding variants (File S1). To gain additional evidence for candidacy, we examined gene expression by RNAseq, using somatosensory cortex and thalamus as tissue source, comparing parent strains B6J and FeJ to each other. In the SWD length 95% CI, of 93 mRNAs expressed, 15 had abundance differences (e.g. q<0.1; File S1). Most were modest (<5% change) but for *Ly6a*, one of several members of a cell membrane protein-encoding gene family, FeJ had a 21% transcript reduction in thalamus and 32% in cortex. Although *Ly6a* is best known for expression in lymphocytes, it is also expressed in brain and at least one Ly6a knockout allele has prenatal lethality [27]. Further refining the *G4swdm1* critical interval, and more extensive testing of existing mutants or creation of others by mutagenesis is required before the correct *G4swdm1* candidate(s) can be identified.

## Discussion

Here we unravel the genetic complexity of *Gria4*-deficiency absence seizure susceptibility in C3H mice. First, we show genetically that *Pcnxl2* deficiency accounts for the unusual substrain difference in spike-wave discharges (SWD) in *Gria4* mutants on C3H/HeJ (HeJ) compared to C3HeB/FeJ (FeJ). We determined that two new frameshift alleles, generated directly in FeJ-*Gria4* ^IAP^ by TALEN mutagenesis, confer SWD mitigation even more than the natural, presumably hypomorphic *Pcnxl2* IAP insertion allele of HeJ. This insertion is of the same IAP-1Δ1 subtype that caused the original *Gria4* ^IAP^ mutation. Given increased incidence and severity of SWD in FeJ-*Gria4* ^IAP^ compared to HeJ-*Gria4* ^IAP^, and the absence of the *Pcnxl2* IAP from two other C3H/He substrains, we think it is reasonable to speculate that *Gria4* ^IAP^ conferred a selective disadvantage to the progenitors of HeJ mice, one that was later diminished upon fixation of the *Pcnxl2* ^IAP^ insertion. These findings also suggest that a number of apparent IAP element differences between C3H substrains, nominally 20% of the IAP-1Δ1 pool, remain a potentially powerful source of genome plasticity, which naturally would not be restricted to neurological phenotypes. Although it was suggested previously that most functional IAP insertions in mouse strains were lost because of deleterious effects, [23], clearly some remain functional – perhaps in the case of *Pcnxl2* due to selective advantage such as the one we hypothesize.

The further phenotypic difference between FeJ and B6J strains highlights the existence of additional genetic influences on *Gria4* associated absence seizures. To begin to dissect these, genome scans revealed several potential modifiers, affecting one or the other SWD measure. By using a congenic strain we validated one modifier locus – *G4swdm1* on Chr 15 and observed its striking effect on SWD length; in F_1_ hybrid mutants, for example, we observed several SWD episodes lasting over 40 seconds. It is difficult to imagine that such frequent and contiguous states of neural hypersynchrony do not have a broader impact on behavior. Despite its clear effect, *G4swdm1* accounts for only some of the phenotype difference between strains – perhaps 30% by comparing SWD length of B6J to F_1_ hybrid (e.g. as illustrated in Figure 4C to 4D). But when multiple interactions are likely, as in complex traits, any such estimates of effect are overly simplistic. Approaches to map such modifiers using conventional quantitative trait locus, especially in small crosses, are limited to loci that show significant main effects despite interactions, or to simple, pairwise interactions when they are quite strong. Novel computational approaches such as CAPE, which incorporates relationships between phenotypic features directly into the gene interaction model [28], may be required to parse more complex interactions in conventional cross designs.

*Pcnxl2* is the first absence seizure modifier gene to be identified in any species, and as such it represents the first of what is likely to be many genetic interactions beneath the complexity of absence seizures. The predicted peptide structure of PCNXL2 is similar to that of other pecanex orthologues, with 8 putative transmembrane domains followed by a conserved so-called pecanex domain. But no known primary or predicted secondary structures have been identified that would predict further function. *D. melanogaster* pecanex (*pcnx*) localizes to the endoplasmic reticulum (ER) and functional genetic studies utilizing the protein unfolded response as a readout, suggest that it shows maternal inheritance and has a role in notch signaling [25]. From conservation among pecanex family members we might expect that *Pcnxl2* is also expressed in the ER. Although much further discussion of function is merely speculation, if it is an ER protein one tempting possibility is a role in trafficking or in posttranslational modification of synaptic receptors such as *Gria4* or other compensating ion channel receptors. The prominent *Pcnxl2* expression in layer V of the cerebral cortex opens the door to the possibility that it is involved in mediating excitatory output from layer V pyramidal neurons to the reticular thalamus and thalamus. Whether any such function is the result of an acute *Pcnxl2* role, or instead a role in circuit development, will require further studies, for example, the creation of a conditional allele to be induced at different ages.

Laboratory mouse strains are well known to vary in susceptibility to experimentally-induced partial or generalized tonic-clonic seizures and there are several well-characterized strain differences modifying the penetrance or severity of so-called monogenic seizure mutations (as discussed earlier) although only one such modifier gene has been identified to date, and interestingly this has also be implicated in human epilepsy [13]. The rate of human gene discovery is rapidly accelerating due to efficient high-throughput exome sequencing [29], but the new progress so far is for syndromic pediatric encephalopathies such as Lennox-Gastaut syndrome. The pace remains much slower for genetic generalized epilepsies, including absence epilepsy. With new mutagenesis technologies such as TALEN and CRISPR to more readily validate candidate genes in large intervals such as those defined in modifier or QTL mapping, the pursuit of modifiers still holds promise for unbiased discovery of new genes, pathways and future novel therapies for idiopathic disease.

## Materials and Methods

### Mouse strain care and sources

All mice were housed and procedures performed with approval of Institutional Animal Care and Use Committee (IACUC). All mice were obtained from The Jackson Laboratory, maintained in a room with a 14 h hour light on/10 h light off cycle, and given free access to LabChow meal and water.

C3H/HeJ (HeJ), C3HeB/FeJ (FeJ), C3H/HeOuJ, C3H/HeSnJ and C57BL/6J (B6J) inbred mouse strains were obtained from The Jackson Laboratory production colonies and subsequently maintained by sib-matings. C57BL/6J.129-*Gria4* ^KO^ congenic knockout mice were originally obtained from Deltagen, Inc, as previously described [16]. FeJ.HeJ-*Gria4* ^IAP^ congenic mice were generated by backcrossing the *Gria4* ^spkw1^ mutation from its original strain, HeJ, to FeJ for 14 generations, bred to homozygosity and maintained by sib-matings. FeJ-*Gabrg2* ^tmSpet^ congenic mice were generated by backcrossing the *Gabrg2* ^tmSpet^ (also known as *Gabrg2* ^R43Q^) knockout mutation from B6J.129-*Gabrg2* ^tmSpet^ congenic mice (originally obtained from Bionomics, LTD) successively for at least 20 generations to FeJ and maintained in the same way. FeJ.B6J-*Scn8a* ^8J^ congenic mice were generated by successive backcrossing of *Scn8a* ^8J^ (also known as *Scn8a* ^V929F^) for at least 15 generations to FeJ and maintained in the same way, as described recently [30]. The 89 backcross mice used for genome-wide mapping were generated by mating (B6J.129-*Gria4* ^KO/KO^×FeJ.HeJ-Gria4^IAP/IAP^)F_1_ hybrids to B6J.129-*Gria4* ^KO/KO^ congenic mice. The 84 B6J-FeJ-*G4swdm1* congenic intercross mice were created by successively backcrossing the FeJ alleles for genetic markers in the critical interval on Chr15 from an N_2_ to the B6J.129 strain while also selecting for *Gria4* ^KO^ homozygotes, and then intercrossing at generation N_9_ or N_10_. The generation of *Pcnxl2* mutants is described below.

### TALEN mutagenesis

We contracted Transposagen Biopharmaceuticals, Inc. to design and construct plasmids for TALENs specific to exon 16 (TALEN A) and exon 29 (TALEN B) of *Pcnxl2*. Target sequences were as follows; TGAGCCGGCAGAGCAGTG and GTGAGTAGCTGTCCTGTA for TALEN A and TATTTGCTGACATGGAC and TTGTTCCAGCCATCCGAA for TALEN B. TALEN plasmids were linearized by *PmeI* endonuclease digestion. One microgram of linearized plasmid was used as a template for in vitro transcription using AmpliCap-Max T7 High Yield Message Maker Kit (CELLSCRIPT) according to the manufactures instruction. A poly(A) tail was added to the synthesized RNA with the A-Plus Poly(A) Polymerase Tailing Kit (CELLSCRIPT) according to the manufactures instruction. The poly(A) tailed capped RNA was purified by ammonium acetate precipitation, resuspended in RNase free water and the concentration determined by spectrophotometry. TALEN mRNA was diluted to 10 ng/υl in RNase free 1X TE (10 mM Tris-HCl, 1 mM EDTA, pH 7.5) immediately before microinjection into embryos obtained from superovulated FeJ.HeJ-*Gria4* ^IAP/IAP^ congenic mice. The genomic DNA made from the tail tip of 63 TALENA mutant founders was amplified with primers aFXTN (5′-CATCGTGGCTGTCGTAATTC -3′) and aRXTN (5′-CATAGCGTGGGAGAGAAAGA-3′). The product was purified and sequenced using primer aF2XTN (5′-GCACACACCACTCATTCATC-3′). The genomic DNA made from the tail tip of 43TALENB mutant founders was amplified with primers bFXTN (5′- GCTTTGTAATGTGGGTTCTG-3′) and bRXTN (5′- GGTTCTCTACTTCAGCCTATG-3′). The product was purified and sequenced using primer bF2XTN (5′-GAACTCGGGATCCATGTTTG -3′).

### Genome scan, interval mapping

For the genome scan, genomic DNA was prepared from tail tips as previously described and sent to Kbioscience (currently LGC Genomics, LLC. Beverly, MA), using a custom single nucleotide polymorphisms (SNP) panel comprised of 187 roughly evenly spaced SNPs. Prior to interval mapping, the raw traits SWD incidence and SWD length were rank-ordered and normal quantile transformed, and from which principal components were derived using JMP software (SAS Institute); once obtained, both principal components were also rank- and normal-quantile transformed, also using tools in JMP. The computer program J/qtl [31] was then used for genome-wide interval mapping of the initial backcross, and for Chr 15 interval mapping of congenic intercross mice. To control for a modest effect (p<0.04) of *Gria4* ^KO/KO^ homozygous null vs *Gria4* ^KO/IAP^ compound heterozygous null/hypomorph genotypes segregating in the N_2_ cross, a marker linked to *Gria4* on Chr 9 was used as a covariate. Sex-averaged genetic map coordinates for SNP markers were obtained from the Mouse Genome Informatics database at The Jackson Laboratory (http://informatics.jax.org). For interval mapping, the multiple imputation model was used and permutation shuffling employed to determine genome-wide significance thresholds.

### Genotyping

C3H/HeJ-*Pcnxl2* ^IAP^ (IAP insertion in intron 19 of C3H/HeJ) was genotyped in standard PCR conditions and agarose gel electrophoresis using one assay for the wild-type allele (primers c8-128.3F 5′-AGCGATGAGGACTGTGGTTT-3′; c8-128.3R 5′-CGAGCCCTTCAGCTACTCAC-3′) and a second assay for the insertion allele (primers c8-128.3F 5′-AGCGATGAGGACTGTGGTTT-3′; IAPLTR5′R 5′-GGCTCATGCGCAGATTATTT-3′) giving a 364 bp product from the insertion allele and 432 bp product from the endogenous allele. The TALEN A+1 or frameshift 1 (FS2) allele was genotyped in standard PCR conditions at an annealing temperature of 64°C and agarose gel electrophoresis using one assay for the wild-type allele (primers Pcnxl2A1WF2 5′-GCAGAGCAGTGATCCTTCAG -3′; Pcnxl2A1WR2 5′-CCATAGCGTGGGAGAGAAAGAA -3′) and a second assay for the mutant allele (primers Pcnxl2A1MF2 5′-GCAGAGCAGTGATCCTTCAC-3′; Pcnxl2A1WR2 5′-CCATAGCGTGGGAGAGAAAGAA -3′) giving a 311 bp product for both alleles. TALEN B-2 or FS3 allele was genotyped in standard PCR conditions with an annealing temperature of 67°C and agarose gel electrophoresis using one assay for the wild-type allele (primers Pcnxl2BwtF 5′-TAGATGCTGGTAGGAGTGAAGA -3′; Pcnxl2BwtR 5′-GGCTGGAACAACAACTTTGTGT-3′) and a second assay for the mutant allele (primers Pcnxl2BwtF 5′-TAGATGCTGGTAGGAGTGAAGA -3′; Pcnxl2BmutR 5′-GGCTGGAACAACAACTTTGTAG-3′) giving a 228 bp product from the mutagenized allele and a 227 bp product from the wildtype allele. TALEN A-11 or FS1 allele was genotyped in standard PCR conditions at an annealing temperature of 55°C and agarose gel electrophoresis using primers Pcnxl2AF2 (5′- CACTGTTCTCGGCCTTCTG-3′) and Pcnxl2AR (5′-AGACATGTGGACATGCGTTTA-3′) giving a 123 bp product from the mutagenized allele and a 134 bp product from the endogenous allele.

### Detection and cloning of IAP-1Δ1 insertions from genomic DNA

For direct detection of IAP-1Δ1 insertions, dried gel hybridization was done essentially as previously described [32] except using an oligonucleotide probe that spans the 1.2 gag-pol common deletion of IAP-1Δ1 elements [22]. Briefly, 8 µg of high quality mouse genomic DNA (obtained from The Jackson Laboratory DNA Resource) was digested with restriction enzyme *Bgl* II, electrophoresed overnight on 0.8% Tris-borate EDTA agarose gels, EtBr-stained and imaged, denatured in NaOH, neutralized and dried for several hours on a flat slab gel dryer with minimal vacuum. 5′-^32^P radiolabeled 31-nt oligonucleotide probe (IAPd1oligo1-R; 5′ ATACCTCTTATCAGGTTCAGCAGAATAAGCTC-3′) was hybridized overnight, the dried gel was washed and imaged on x-ray film. For cloning of IAP-1Δ1-host junction fragments by inverse PCR: 1 µg of genomic DNA was digested with *Bgl*II, diluted, then 10 ng was ligated for 2 hrs at room-temperature using T4 DNA ligase, heat-inactivated, then circular product was amplified in a polymerase chain reaction (PCR) using an oligonucleotide that spanned the IAP-1Δ1 deletion (IAPd1oligo2F, 5′- GAGCTTATTCTGCTGAACCTGATA-3′) paired with an oligonucleotide specific for the IAP LTR (IAPLTR5′, 5′- GGCTCATGCGCAGATTATTT-′3). Amplified fragments were visualized by agarose gel electrophoresis (e.g. Figure 1C), excised and extracted from agarose using Qiagen minicolumns, cloned into Bluescript plasmid by T/A cloning (Invitrogen), transformed into a suitable *E. coli* K12 host, miniprepped and subjected to Sanger sequencing using T7 and Sp6 vector primers.

### Quantitative RT-PCR

Total RNA was prepared from the whole brain of adult HeJ, FeJ, and FeJ-*Gria4* ^IAP^ and the hippocampus of adult FeJ- *Gria4* ^IAP^ either wildtype or carrying *Pcnxl2* TALEN mutations with Trizol (Invitrogen) and treated with DNase I (Promega) under the manufacturer's suggested conditions. RNA (2 µg) was reverse transcribed with AMV reverse transcriptase (Promega). The cDNA was diluted 20-fold, and 2 µl was added to DyNAmo HS SYBR Green qPCR master mix (Thermo Scientific) with pairs of the following primers; *beta-actin*F (5′- ATGCTCCCCGGGCTGTAT-3′) and *beta-actin*R (5′- CATAGGAGTCCTTCTGACCCATTC-3′), *Pcnxl2*exon19F (5′-GGATCTCACATCCTGTGCTC -3′) and *Pcnxl2*exon20R (5′-CCACACGTAGAGTCTCTCAAAC -3′), *Gria4*exon15F (5′- GGTGGCTTTGATAGAGTTCTGTTACA-3′) and *Gria4*exon16R (5′- TCTTATGGCTTCGGAAAAAGTCA -3′), *Pcnx*exon35F (5′-GAACAGCTGGAAAGACTGGA-3′) and *Pcnx*exon36R (5′-CGATGTGGGACCTTGTACTT-3′). The PCR reactions were analyzed on an Applied Biosystems 7500 Real-Time PCR System. The PCR amplifications from three mice of each strain and/or genotype were run in triplicate. Amplification of the correct size products was confirmed by agarose gel electrophoresis. The ΔΔΧt method was adopted for the calculation of relative transcript levels.

### mRNAseq

Somatosensory cortex or thalamus was dissected from wildtype or *Scn8a* ^8J/+^ B6J and FeJ adult male mice in triplicate, and prepared for high-throughput sequencing on the Illumina HiSeq 2000. The Jackson Laboratory Gene Expression Service prepared mRNA sequencing libraries using the Illumina TruSeq methodology. Tissue was placed in RNALater (Qiagen, Inc, MD), RNA was extracted using TRIzol (Invitrogen, CA). For mRNA-Seq, mRNA was purified from total RNA using biotin tagged poly dT oligonucleotides and streptavidin coated magnetic beads followed by quality control using an Agilent Technologies 2100 Bioanalyzer (Agilent Technologies, Santa Clara, CA, USA). The mRNA was then amplified and double-stranded cDNA was generated by random priming. The ends of the fragmented DNA were converted into phosphorylated blunt ends. An ‘A’ base was added to the 3′ ends. Illumina-specific adaptors were ligated to the DNA fragments. Using magnetic bead technology, the ligated fragments were size selected and then a final PCR was performed to enrich the adapter-modified DNA fragments since only the DNA fragments with adaptors at both ends will amplify. The sequencing library was first validated using an Agilent Technologies 2100 Bioanalyzer to characterize DNA fragment sizes and concentration. The concentration of DNA fragments with the correct adapters on both sides was then determined using a quantitative PCR strategy, following the kit manufacturer's protocol (Kapa Biosystem, Cambridge, MA). Following library quantitation, libraries were diluted and pooled as necessary. Using the Illumina cBot, libraries were added to the flow cells and clusters were generated prior to 100 bp paired end sequencing on the Illumina HiSeq 2000 (Illumina, San Diego, CA, USA). During and after the sequencing run, sequence quality was monitored using the real time analysis (RTA) and sequence analysis viewer (SAV) software available by Illumina. Following sequencing, demultiplexed fastQ files were generated using the Illumina CASAVA software.

FastQ files were aligned to the C57BL/6J reference genome on a high performance computing cluster using Tophat (http://tophat.cbcb.umd.edu/) for the alignment and RSEM (http://deweylab.biostat.wisc.edu/rsem/) for isoform assembly and quantitation, except that frequency of reads per kilobase was normalized based on quartile instead of the total number of mapped reads. Further analysis was done in R (http://www.R- project.org) using ANOVA and linear modeling to test expression differences by strain, genotype and tissue, and FDR analysis was done in Microsoft Excel (Microsoft Corp).

### EEG recording and analysis

Adult mice aged between 6 and 8 weeks were anesthetized with tribromoethanol (400 mg/kg i.p.). Small burr holes were drilled (1 mm anterior to the bregma and 2 mm posterior to the bregma) on both sides of the skull 2 mm lateral to the midline. Four teflon-coated silver wires were soldered onto the pins of a microconnector (Mouser electronics, Texas). The wires were placed between the dura and the brain and a dental cap was then applied. The mice were given a post-operative analgesic of carprofen (5 mg/kg subcutaneous) and allowed a minimum 48 h recovery period before recordings. Differential amplification recordings were recorded between all four electrode pairs, providing 6 channels for each subject. Mice were connected to the EEG Stellate Lamont Pro-36 programmable amplifier (Lamont Medical Instruments, Madison, WI) for a 2-hour period on 2 separate days, between the hours of 9 AM and 4 PM during the lights-on period. EEG data were recorded with Stellate Harmonie software (Stellate Systems, Inc., Montreal, Canada) into a database. SWD consist of adjacent, connected spike-wave (or wave-spike) complexes. Recordings were reviewed using low/hi bandpass filters at 0.3 Hz and 35 Hz respectively, and SWD episodes were scored blinded to genotype using the following criteria: at least 2 connected spike-wave complexes (typically spanning at least 0.5 seconds) with amplitudes at least two fold higher than background and observed concurrently in the majority of the 6 recording channels per mouse.

## Supporting Information

File S1 A MS Excel workbook pertaining to the *G4swdm1* modifier interval on Chr 15, with five worksheets it in including: 1. List of all genes in the *G4swdm1* critical interval (JAX-MGI). 2. List of all sequence variants between B6J and HeJ in the interval (Sanger-MGP). 3. List of possibly functional/deleterious variants in the interval (Sanger-MGP). 4. RNAseq results from B6J and FeJ thalamus and somatosensory cortex for Chr 15. 5. Subset of RNAseq results for genes in the *G4swdm1* critical interval.
(XLSX)
